# Whole-genome sequencing and phylogenetic analysis of coxsackievirus-A16 strains causing hand, foot and mouth disease (HFMD) in India

**DOI:** 10.1099/mgen.0.001130

**Published:** 2023-10-31

**Authors:** Sanjaykumar Tikute, Sanket Sonawane, Anita Shete, Abhinendra Kumar, Savita Yadav, Pragya D. Yadav, Mallika Lavania

**Affiliations:** ^1^​ Enteric Viruses Group; ICMR-National Institute of Virology, Pune, Maharashtra, India; ^2^​ Maximum Containment Laboratory, ICMR-National Institute of Virology Pune, Pune, Maharashtra, India

**Keywords:** Coxsackievirus A16, Functional genomics, HFMD, Myocarditis, Phylogenetic analysis

## Abstract

Hand, foot and mouth disease (HFMD) is a common childhood infectious disease, caused by enteroviruses (EVs), which can present with typical or atypical lesions. The illness is self-limiting, but it can also have serious complications. Since 1997, HFMD infections have become endemic and have increased to epidemic proportions across the Asia Pacific region, including India. Coxsackievirus-A16 (CV-A16) outbreaks occurred in India from 2005 onwards, although the clinical symptoms were noticeably different during this period. Understanding the population dynamics of enteroviruses that cause HFMD is crucial in the post-polio era because one of the circulating strain may replace another as the dominant strain. The aim of this study is to describe the genetic features of the CV-A16 strains isolated from hand, foot and mouth disease (HFMD) patients in India. Reverse transcription PCR (RT-PCR) and cell-culture-based isolation of CV-A16 was done from the 55 clinical samples. The entire genome of the CV-A16 isolate was performed from the seven isolates. After the sequences were analysed, a phylogenetic tree was created using bioinformatics tools. The total genomic length obtained was 7411 base pairs (bp). Nucleotide similarity across various regions, including 5′UTR, P1, P2 and 3′UTR, ranged from 87.0–97.9 %, 77.0–95.4 %, 80.3–96.9 %, and 77.9–96.2 %, respectively. Correspondingly, similarities in the VP1 region’s nucleotide and amino acid sequences were 91.4–96.4 % and 99.3–99.7 %, respectively. Phylogenetic analysis highlighted that CV-A16 strains identified in Pune, Maharashtra, were grouped within the same cluster. The analysed CV-A16 isolates in this study aligned with subgenotype B1c. These findings have far-reaching implications for the surveillance, prevention and management of HFMD and CV-A16. Monitoring the dynamics of CV-A16 strains, informed by the genetic characteristics identified here, will significantly impact strategies aimed at tackling HFMD and its associated public health challenges.

## Data Summary

The genome sequences generated in this study submitted to NCBI. The genome assembly with annotation deposited in the NCBI GenBank under accession numbers (OR437333–OR437338). The authors confirm all supporting data, code and protocols provided within the article.

**Impact Statement**

This study highlights the application of next-generation sequencing, instead of Sanger sequencing, in distinguishing between coxsackievirus strains. Since 2005, CV-A16 has gradually become the predominant pathogen responsible for HFMD in several parts of the India. Because the number of HFMD cases with generalized skin lesions has increased in India, we performed molecular identification of enteroviruses from HFMD cases, virus isolation from these clinical specimens, whole-genome sequencing and phylogenetic analysis, as well as a determination of clinical relevance. Molecular characterization was done for circulating CV-A16 strain and it was found out to be CV-A16 of sub-genotype B1c. Full genome was analysed using CV-A16 previous reference strains. Analysis of VP1 gene revealed several nucleotide changes and amino acid substitutions.

## Introduction

Hand, food and mouth diseases (HFMD) is a paediatric disease occurs usually in children under the 5 year age group [1]. The clinical manifestations of HFMD include mild to severe skin rash, pulmonary oedema, circulatory disorders, meningoencephalitis, aseptic encephalitis, and even death. It has been shown that there have been significant outbreaks in the Asia-Pacific area since 1997 [2, 3]. Large-scale HFMD outbreaks have been reported in China during the past few years [4–6]. The first HFMD outbreak in India was identified in Calicut, Kerala, in 2005 [7]. Subsequently, multiple serotypes related outbreaks reported from West Bengal, Odisha, Tamil Nadu, and Karnataka [8–10]. Due to low suspicion, the fact that common illnesses like chicken pox and mosquito bites have similar clinical presentations and can lead to misdiagnosis, or the fact that the disease is typically self-limiting with uneventful recovery, the disease was relatively unknown in India until recently, which led to an underestimated disease burden [8, 11].

The etiological agents associated with the viral infection are found to be coxsackievirus-A6 (CV-A6), CV-A10, CV-A16 and enterovirus-A71 (EV-A71). The world-wide clinical survey revealed that both the viral strains EV-A71 and CV-A16 could contribute to cause neurological manifestations and fatalities [12]. CV-A16, CV-A6, CV-A10 and EV-A71 were the major etiological agents of HFMD in India, the data on recently circulating enteroviruses associated with HFMD are sparse [9, 13–15].

The mature viral capsid proteins P1 (VP4, VP2, VP3 and VP1), as well as the non-structural proteins P2 (2A to 2C) and P3 (3A to 3D), are all encoded by a single open reading frame in the EV genome. Enteroviruses produce genetic variety and evolve by genetic recombination or nucleotide mutation. According to epidemiologic studies distinct enteroviruses have been known to recombine with each other [5, 16, 17]. There was no concrete proof that inter-typic recombinant EVs had previously contributed to HFMD outbreaks [5], but severe or fatal cases of HFMD were reported to be caused by inter-typic recombinant EVs [17, 18]. Historically, the two main etiological agents of HFMD reported globally were EV71 and CV-A16 [19].

Since 2005, CV-A16 has gradually become the predominant pathogen responsible for HFMD in several parts of the India. Because the number of HFMD cases with generalized skin lesions has increased in India, we performed molecular identification of enteroviruses from HFMD cases, virus isolation from these clinical specimens, whole-genome sequencing and phylogenetic analysis, as well as a determination of clinical relevance.

## Methods

### General information about our samples and the HFMD cases

Sporadic and outbreak cases of HFMD were reported during the years 2013–2022 from Pune and other parts of Western India. In the study, 565 clinical specimens, which includes throat swabs, vesicular swabs and stool specimens, were collected from HFMD patients. An HFMD outbreak caused by both CV-A16 and CV-A6 was also observed last year in 2022. All the patients belong to the paediatric age group ranged between 1 month to 10 years old. Clinical features of the disease included rashes on hand, feet, buttocks along with mouth ulceration with preceding mild febrile illness. All demographic details were recorded in well-defined case reporting forms (CRF). Prior written informed consent was taken from the parents/guardians prior to sample collection. Three hundred and nineteen of these 565 cases tested positive for enterovirus by PCR.

### Ethical approval

The Institutional Ethics Committee of ICMR-National Institute of Virology, Pune, approved this work (No: 23-1-8M).

### Clinical specimens

Patients who met the following three criteria were selected for clinical correlation analysis: (1) under 10 years of age, (2) skin lesions compatible with typical HFMD or atypical HFMD, (3) CV-A16 identified by virological methods. The skin lesions of typical HFMD are commonly manifest as small vesicles, papulovesicular lesions or macular rashes on the palms, soles, buttocks and oral mucosa. Atypical HFMD is manifest as large vesicles or bullae, maculopapular rashes or target-like lesions presenting on any site of the body including the trunk, limbs or facial areas, as well as symptoms of acute viral infection, such as fever, cough or diarrhoea.

Clinically positive samples of HFMD from different regions of India were collected and referred from hospitals and paediatric clinics to the Enteric Viruses Group (EVG) at ICMR-National Institute of Virology, Pune. Clinical specimens included throat swabs (236), vesicular swabs (315), stool samples (17), rectal swab (46) and others (62) from HFMD patients (Table 1) were collected during the three outbreaks.

**Table 1.** Types of samples collected at outbreaks occurring during different years

	2013–2014	2017–2018	2021–2022	Total
**Throat swabs**	62	39	135	236
**Vesicular swabs**	40	50	225	315
**Rectal swabs**	46	0	0	46
**Stool**	10	4	3	17
**Oral lesion swabs**	0	0	6	6
**Urine**	0	0	40	40
**Serum**	0	0	16	16
**Total**	158	93	425	676

Seven isolates with Ct value <25 will include in the study were from the vesicular swabs of the clinically and molecularly confirmed HFMD positive patient from Pune (western region of Maharashtra state) region at different time periods.

### Serotypic identification of enteroviruses from the clinical specimens

Viral RNA was extracted directly from clinical specimens using a QIAamp Viral RNA Mini Kit (Qiagen, Santa Clara, CA) and stored at −80 °C. The detection of enterovirus from the isolates was carried out by RNA extraction followed Q-PCR using the Pan Enterovirus (EV) real-time reverse-transcription PCR (rRT-PCR) using specific primers [20]. As recommended by the manufacturer (Invitrogen, Life Technologies), single complementary DNA (20 ul reaction) was synthesized and used for EV genotyping of EVs. VP1 and VP1/2A gene junction areas were amplified by using nested RT-PCR to determine the EV genotypes [21, 22]. Genotyping and sub-genotyping were performed on all of the positive samples. The whole VP1 gene was amplified using the previously published semi-nested genogroup specific PCR [22]. Briefly, 50 µl of the master mix was prepared to contain 10X PCRbuffer, 10 mM dNTPs, Forward and reverse primers (20 pmoles/μl), taqDNA polymerase (5 U/µl Roche, Germany) and the final volume wasmade up with distilled water (MilliQ water; Bangalore Genie Pvt Ltd India). EV serotypes were identified by using semi-nested RT-PCR and sequencing as previously described [22]. Serotype was determined by comparison of the viral sequences with corresponding sequences of the EV prototype strains using blastn online (http:// blast.ncbi.nlm.nih.gov/ Blast.cgi).

### Virus isolation

Fifty five molecularly characterized CV-A16 clinical samples were further subjected for virus isolation in Rhabdomyosarcoma (RD) a susceptible cell line [23, 24]. The 100 µl of clinical specimens (mainly vesicular swab and throat swabs) were inoculated onto RD cell line seeded in 24-well plate and incubated at 37 °C, supplement with 5 % CO_2_ for 5–6 days. After incubation, the plate was observed daily for any morphological changes in the cell line. Samples showing cytopathic effects (CPE) were recognized by the cell death due to viral infection. CPE showing cultures were further passaged up to passage level three and viral agent was isolated [25, 26]. Confirmation of the viral isolates was further carried out by VP1 gene amplification.

### Whole-genome sequencing of CV-A16 isolates

Seven-isolated virus CV-A16 from different time periods were subjected for full genome sequencing using next-generation sequencing illumina platform. RNA Extraction was performed using QIAmp Viral RNA extraction kit (Qiagen) as per the manufacturer’s protocol. The RNA concentration was quantified by Qubit 2.0 Fluorometer (Invitrogen, Life Technologies) using Qubit RNA High Sensitivity (HS) kit and then stored at −20 ˚C until use. The host ribosomal RNA was depleted using NEBNext rRNA depletion kit (New England Biolabs) (Human/mouse/rat). This RNA was further purified using Agencourt AMPure XP beads (Beckman Coulter). The depleted RNA was quantified with Qubit RNA High Sensitivity (HS) kit. The RNA library was prepared using the TruSeq Stranded mRNA LT Library preparation kit Illumina), which involve rRNA depletion, fragmentation, amplification and Qubit quantification. The amplified libraries were quantified, normalized and loaded on the Illumina Miniseq platform. After the completion of the run, FASTQ files were imported and analysed using CLC Genomics Workbench software version 22.0.4 (CLC, Qiagen). The reference-based assembly method in the workbench was used to retrieve the coxsackievirus-A16 sequences. MG957117 was used as reference for CV-A16 strain (accession ID: KM215267).

The evolutionary history was inferred using the neighbor-joining method [27, 28]. The bootstrap consensus tree inferred from 1000 replicates [29] is taken to represent the evolutionary history of the taxa analysed [29]. Branches corresponding to partitions reproduced in less than 50 % bootstrap replicates are collapsed. The percentage of replicate trees in which the associated taxa clustered together in the bootstrap test (1000 replicates) are shown next to the branches [29]. The evolutionary distances were computed using the maximum composite likelihood method [30] and are in the units of the number of base substitutions per site. The rate variation among sites was modelled with a gamma distribution (shape parameter=0.5). This analysis involved 69 nucleotide sequences. All ambiguous positions were removed for each sequence pair (pairwise deletion option). There were a total of 7471 positions in the final dataset. Evolutionary analyses were conducted in mega11.

## Results

### Isolation of CV-A16 strains

**Fig. 1.**
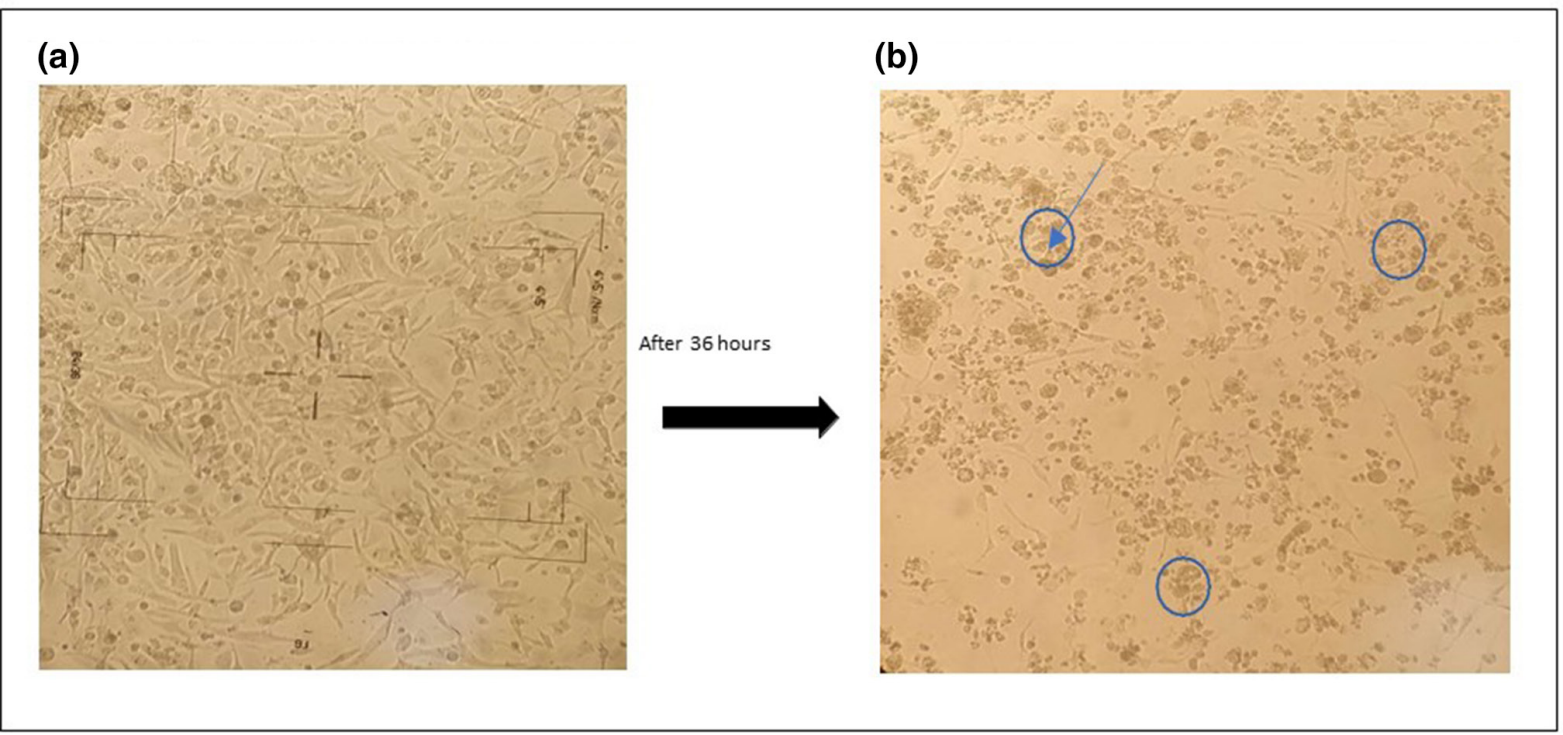
Isolation of virus on RD cell lines.

For virus isolation, 55 specimens were inoculated into the human rhabdomyosarcoma (RD) cell line and cultured for up to three passages [28] (Fig. 1).

Monolayer RD cells line (left 1A) and cells infected with clinical specimen (right 1B). Morphological changes are observed after infection

Pathogens other than enteroviruses were not investigated. The statistical differences between proportions were tested using the Chi-square test or Fisher’s exact test.

### Serotyping of enteroviruses associated with HFMD cases circulating in Pune, India

**Fig. 2.**
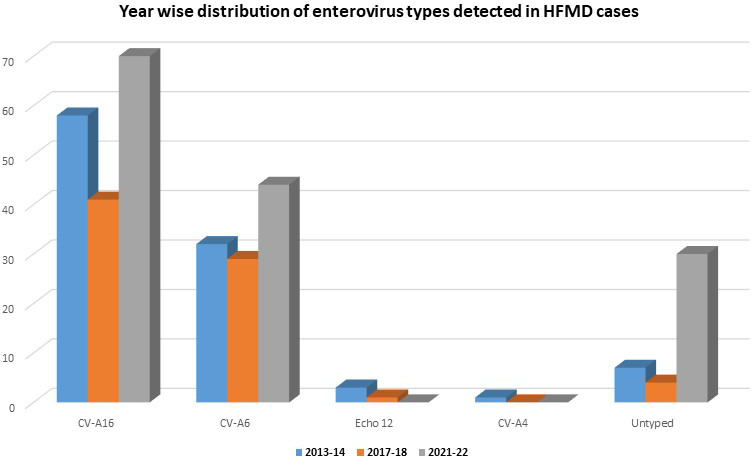
Year wise distribution of genotypes of EVs detected in HFMD cases.

A total of 676 patients with a clinical diagnosis of HFMD were investigated in this study. A definite serotype was identified in 279/320(87.2 %) cases and the most common EV-A was CV-A16, which accounted for 169(52.8 %) cases, followed by CV-A6 in 105(32.8 %) (Fig. 2).

### Full-length genomic sequencing of the recently emerging CV-A16 isolates

In order to determine the molecular characteristics of the predominant CV-A16 strains responsible for the outbreak of CV-A16-associated HFMDs, seven CV-A16 strains with good CPE from 07 cases before and during the CV-A16 outbreak from 2013 to 2022 were selected, and the entire genome of each virus was amplified directly from the virus isolate. Out of seven isolates, we could retrieve a total of six isolates above 99.0 % genome (Table 2).

**Table 2.** Overview of NGS CV-A16 sequences obtained from seven isolates using Illumina sequencing

Mcl id (NIV ID)	Age/sex	Clinical symptoms	Total reads	Genotype(VP1) assignment	Genome coverage	Consensus length	Isolation year
MCL-22-H-2380 (CV-A16/588)	3 years/M	Fever, rashes on hand and tongue	12,31,730	CV-A16	99.89 %	7400	2013
MCL-22-H-2379 (CV-A16/907)	1.5 years/F	Fever, rashes on knees and feet	11,05,296	CV-A16	99.91 %	7401	2013
MCL-22-H-2382 (CV-A16/309)	2.3 years/M	Fever, rashes on hand and feet	7,94,944	CV-A16	99.82 %	7395	2013
MCL-22-H-2378 (CV-A16/311)	3 years/F	Fever, rashes on hand, feet and ulcers in mouth	9,15,128	CV-A16	99.89 %	7400	2018
MCL-22-H-2381 (CV-A16/170)	2.5 yrs/M	Fever, rashes on hand and knees	9,73,984	CV-A16	99.82 %	7395	2018
*MCL-22-H-7446 (CV-A16/121)	3.5 years/F	Fever, rashes on hand and buttocks	2,01,792	CV-A16	42.85 %	3174	2022
MCL-22-H-7447 (CV-A16/123)	3.5 years/F	Rashes on hand and feet	1,76,920	CV-A16	99.08 %	7340	2022

*Genome coverage was not good so was excluded from the analysis.

The case ID MCL-22-H-7446 retrieved 42.85 % (relevant reads) of genome because of low viral load while MCL-22-H-7447 showed high viral load with retrieval of 99.08 %. Total reads reveals the presence of any microflora of human including infectious pathogens.

The sequences retrieved in this study were used to construct the phylogenetic tree to confirm the genotype and similarity of the virus. The retrieved sequences (*n*=06) were aligned in CLC genomics software with the representative sequences of CV-A16-specific genotype downloaded from NCBI. Genotype A (*n*=02), genotype C (*n*=10) and genotype B {B1a (*n*=18), B1b (*n*=17) and B1c (*n*=16)}. The aligned sequences were checked and corrected manually in mega 11.0 software.

**Fig. 3.**
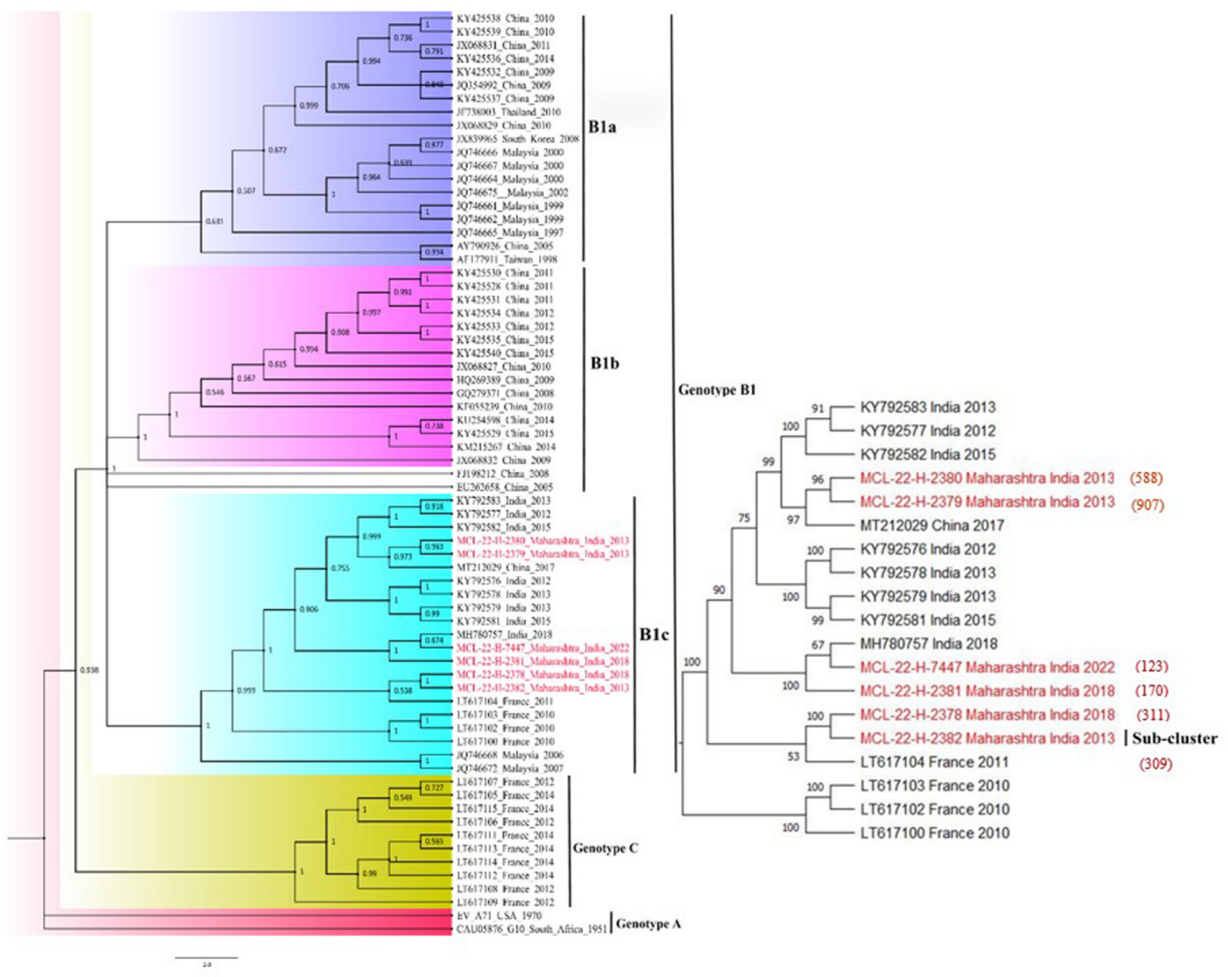
Phylogenetic tree constructed based on the full genome of CV-A16 strains detected in HFMD patients.

The neighbor-joining tree was generated in mega using the Tamura-3-parameter model with a bootstrap replication of 1000 cycles. The retrieved sequences belong to sub-lineage B1c of genotype B1. Out of five sequences, two sequences (MCL-22-H-2380/588 and MCL-22-H-2379/907) showed closed similarity with China 2017 (MT212029), one sequence (MCL-22-H-2381/170]) with India 2018 (MH780757) while the rest two sequences with MCL-22-H-2378/311 and MCL-22-H-2382/309] formed a separate sub-cluster in B1c lineage and showed close similarity with 2011 sequence from France (Fig. 3).

Except for one sample, all the identified CV-A16 strains in the study had 7400 bp of genome length, according to nucleotide sequencing. There were 2197 amino acids. With 7 % nucleotide substitution in the VP1 gene and 93.60 % similarity to the reference strain (AM292476), the isolate CV-A16/170 had the highest level of nucleotide substitution among the chosen samples. Other isolates CV-A16/311, CV-A16/588, CV-A16/907, CV-A16/309, and CV-A16/170 were 95 %, 94 %, 94.73 %, and 95.17 % similar to the reference strain (AM292476), respectively. Amino acid changes in the strain CV-A16/170 in VP1 region occurred which were 2440 in (P3S), 2464 (T11A), 2866 (V145E), 2932 (D167G). The isolates CV-A16/309 (P3S), 2866 (V145E), CV-A16/311 (P3S), 2506 (V18I), CV-A16/588 (P3S) and CV-A16/907 (P3S), respectively, all showed the presence of amino acid substitution (Table 3).

**Table 3.** Amino acid change and nucleotide substitution identified of CV-A16 isolates

NT position on full genome	Cv-a16/170 CV-A16/309 CV-A16/311 CV-A16/588 CV-A16/907									
	Amino acid	Nucle otide	Amino acid	Nucle otide	Amino acid	Nucle otide	Amino acid	Nucle otide	Amino acid	Nucle otide
**2440**	P 3 s	C-T	P 3 s	C-T	P 3 s		P 3 s		P 3 s	–
**2464**	T 11 A	A-G						T-C		
**2475**		–		T-C		T-C		–		–
**2490**		–		–		–		C-T		C-T
**2505**		A-G		–		–		–		–
**2506**		–		–	V 18 I	G-A		–		–
**2508**		–		–		–		A-G		A-G
**2514**		C-T		–		–		C-T		C-T
**2547**		–		–		–		A-G		–
**2548**		–		T-C		T-C		–		–
**2556**		–		–		–		T-A		T-A
**2559**		C-T-		–		–		–		–
**2565**		A-G		–		–		–		–
**2571**		A-T		A-G		A-G		–		A-T
**2601**		T-A		–		–		–		–
**2631**		T-C		–		–		T-C		T-C
**2640**		G-A		G-A		G-A		–		–
**2643**		G-A		–		–		–		–
**2649**		T-C		–		–		T-C		T-C
**2655**		C-T		–		–		–		–
**2697**		T-C		–		–		–		–
**2712**		T-C		–		–		–		–
**2730**		T-C		–		–		–		–
**2742**		–		–		–		A-G		–
**2748**		–		T-C		T-C		–		–
**2758**		T-C		–		–		–		–
**2806**		T-C		–		–		–		–
**2826**		–		–		–		–		T-C
**2853**		–		C-T		C-T		C-T		C-T
**2866**	V 145 E		V 145 E				V 145 E		V 145 E	
**2867**		T-A		T-A		–		T-A		T-A
**2889**		–		–		–		–		G-A
**2904**		A-T		–		–		–		–
**2916**		–		G-A		G-A		–		–
**2933**		A-G		–		–		–		–
**2932**	D 167 G									
**2949**		–		–		–		–		G-A
**3003**		G-A		–		–		–		–
**3027**		–		C-T		C-T		–		–
**3033**		A-T		–		–		A-T		A-T
**3054**		T-A		–		–		–		–
**3072**		G-A		–		–		G-A		G-A
**3126**		–		C-T		C-T		–		–
**3144**		A-G		–		–		–		–
**3156**		G-A		–		–		–		–
**3159**		–		G-A		G-A		–		–
**3180**		–		–		–		G-A		G-A
**3219**		C-T		–		–		C-T		C-T
**3255**		G-A		–		–		–		–
**3270**		–		–		–		–		T-C
**3322**		–		–		–		T-C		–

## Discussions

Enterovirus typing and surveillance is important to track epidemiological trends and potentially link clinical presentation. There are more than 120 different strains of enteroviruses worldwide [1]. However, some infections can cause serious morbidity and mortality [31]. Most patients initially appear with moderate, asymptomatic respiratory or gastrointestinal (GI) symptoms. Human-infecting enteroviruses are divided into enterovirus groups A through D, and they are usually identified during Sanger’s sequencing by focusing on the viral protein 1 (VP1) gene on the viral capsid [32]. Group-A of enteroviruses (EV-A) consists of a total of 23 variants [1].

It can be difficult to genotype enteroviruses using traditional targeted techniques because of their enormous genetic diversity [23, 33, 34]. As an addition to more conventional targeted methods for genotyping, NGS and viral enrichment were used to improve resolution, offer more thorough phylogenetic analysis, and complement traditional targeted approaches.

In the present study, virus isolation is done from the clinical specimens of HFMD confirmed cases during year 2013, 2018 and 2022. Full genome sequences of the isolates were compared with the other reference strains of CV-A16 available in the database. VP1 gene is considered to be the most informative and robust region for evolutionary study due to a high degree of diversity and lack of involvement in recombination [35]. Previously, virulence and pathogenicity sites have been reported on VP1 and VP2. Mutations on R51K, K52R, M98T, N102D, T103A, N218D, E241K, T248A/I, V251L and T/H295A in the VP1 gene can decrease the virulence and pathogenicity of CV-A16 in mice [36].

The evolution of HEVs occurs through genetic drift and, over much longer periods, antigenic diversification in the structural gene region encoding the virus capsid (including VP1). Nucleotide changes and amino acid substitutions in VP1 gene (891 bp) of study isolates CV-A16/170 with four other previous isolates and references strains of VP1 sequences were used to construct the phylogenetic tree.

Through comprehensive analysis of whole genome and VP1 gene sequencing, as well as phylogenetic investigations, the study identified the existence of B1c subgenotype CV-A16 strains. These strains were observed to be circulating within the years 2013 to 2022. This study strains showed 91.58–99.67 % nucleotide and 98.65–100 % amino acid identity with B1c sub-genotype with reference strain from other countries.

In summary, our study demonstrates that CV-A16 is an endemic HFMD pathogen with a single sub-genogroup B1c circulating in India during 2013–2022. Despite its moderate evolution rate and stable genetic diversity, CV-A16 was dispersed widely in Indian population during the study period. Viral circulation in Indian happened some time in 2005, 8 years prior to its detection in HFMD cases enrolled in the present study from 2013 onward. These collective findings emphasize the importance of active surveillance for viral circulation in HFMD endemic countries, critical to informing outbreak response. The VP1 region’s partial nucleotide sequences, which are frequently employed for genotyping and as the basis of phylogenetic analyses, would have only been able to identify one of the 20 nucleotide differences without any variation in the amino acid sequence among the six viral sequences that have been characterized. In-depth genetic investigations are now possible because to the NGS approach utilized in the study, which generates substantially longer nucleotide sequences for each CV-A16 strain (about 7000 bp) including the full VP1 gene. The methodology used in this study is a useful tool in order to develop and compare CV-A16 viral genomes to find out if any variation occurred during the years. For any future research examining enteroviral phenotypic factors and tracking the establishment of changed pathogenicity and tropism, these complete whole-genome sequences will be a valuable resource.

## Institutional review board statement

The study was approved by the Institutional Ethics Committee of ICMR-National Institute of Virology, Pune, Maharashtra, India for studies involving humans.
